# Pulmonary Valve Replacement Timing Following Initial Tetralogy of Fallot Repair: A Systematic Review

**DOI:** 10.7759/cureus.49577

**Published:** 2023-11-28

**Authors:** Ethan Slouha, Genevieve Trygg, Abdul Hadi Tariq, Anthony La, Allison Shay, Vasavi R Gorantla

**Affiliations:** 1 Anatomical Sciences, St. George's University School of Medicine, St. George's, GRD; 2 Biomedical Sciences, West Virginia School of Osteopathic Medicine, Lewisburg, USA

**Keywords:** transannular patch, pulmonary regurgitation, tetralogy of fallot, pulmonary valve replacement, tetralogy of fallot repair

## Abstract

Pulmonary valve replacement (PVR) is a critical aspect of surgical management for patients with tetralogy of Fallot (ToF). Determining an optimal timeframe for intervention is imperative, as it directly impacts long-term outcomes and the risk of complications in ToF patients. Ventriculotomy with the transannular patch is currently indicated for right ventricular outflow tract obstruction, but the patch itself can lead to pulmonary regurgitation (PR), dyspnea, and cyanosis, among other complications. This investigation seeks to establish an evidence-based timeline to enhance the overall quality of care for individuals with this congenital heart condition. From 2002 to 2022, 21,935 articles regarding the PVR timing for ToF were examined and filtered. The publications were screened using PRISMA guidelines, and 32 studies were included for analysis and review. Among the studies, PVR was strongly indicated for patients who had developed severe PR, especially in asymptomatic patients and those experiencing fatigue and exercise intolerance. Severe PR was associated with arrhythmias such as right bundle branch block, atrioventricular block, and prolonged QRS intervals, in which male sex and high right ventricular end-diastolic volume (RVEDV) were significant predictors of long preoperative QRS duration. Most physicians found RVEDV necessary for making surgical referrals despite a lack of correlation between PR severity and RVEDV or indexed right ventricular end-systolic volume (RVESVi). However, asymptomatic ToF patients with preoperative RVESVi benefited from PVR. Except for some variations in QRS intervals among studies, arrhythmias tended to persist post-op, yet NYHA functional class and RV size improved significantly following PVR. Older age at PVR was found to be associated with adverse cardiac events, whereas early PVR presented with appropriately short QRS intervals. Cardiac function tended to be significantly worse in patients undergoing late PVR versus early PVR, with timelines ranging from one to three decades following initial ToF repair. Choosing the best timeline for PVR largely depends on the patient's baseline cardiopulmonary presentation, and additional quantitative deformation analysis can help predict an appropriate timeline for ToF patients.

## Introduction and background

Tetralogy of Fallot (ToF), or Steno-Fallot tetralogy, was one of the first and most common cyanotic lesions described, with an incidence of 0.34 per 1000 live births [1]. It was first described in 1673 by Dne Niels Stensen, an anatomist who essentially paved the way for understanding other congenital heart conditions [1-3]. ToF consists of severe pulmonary stenosis, right ventricle (RV) hypertrophy, overriding aorta, and ventricular septal defect [4]. Pulmonary stenosis arises due to the involvement of the subpulmonary infundibulum, where the third name, "the monology of Stensen," was coined [3]. Detection following birth, including transesophageal echocardiogram and pressure tracings, may be made immediately based on severity or following months due to signs such as cyanosis following exercise or relief of cyanosis by squatting [4]. Failure to recognize ToF can lead to a moribund infant presenting with profound hypoxemia caused by myocardial dysfunction with reduced cardiac output [4]. The optimal repair must be suitable for children of any age and primarily provide appropriate relief of the right ventricular outflow tract (RVOT) obstructions and prevent further RV hypertrophy [2].

Surgery was found to be the most optimal in treating ToF and was first explained in 1955 by Lillehei et al. and centered around relieving the RVOT obstruction [1]. Currently, the RVOT is approached via a ventriculotomy encroaching into the right RV anterior wall, and the obstruction is relieved through a transannular patch if needed [1,5]. Unfortunately, it has become clear that using a transannular patch leads to the development of pulmonary regurgitation (PR) following the primary repair [5]. Pulmonary regurgitation can result in further complications such as chest pain, difficulty breathing, especially during exercise, swelling of the legs, and even cyanosis [6]. Eventually, this can lead to RV hypertrophy, and preventing this complication is one reason for the initial ToF repair [6]. These outcomes are not immediate and occur many years after persistent pulmonary regurgitation, making identification of a basic timeframe for intervention imperative before complications worsen. This investigation aims to determine the possibility of a set time interval for pulmonary valve replacement (PVR) to prevent deterioration of a patient who has undergone a complete tetralogy of Fallot repair.

## Review

Methods

A thorough literature search was performed using PubMed, ScienceDirect, and ProQuest databases from January 1, 2002, to December 31, 2022. Search methods closely adhered to the Preferred Reporting Items for Synthetic Reviews and Meta-analyses protocol [7]. Keywords included 'pulmonary valve replacement timing tetralogy of Fallot' and 'pulmonary valve replacement after tetralogy of Fallot'. The following criteria were used for inclusion: investigations conducted on humans, focus on pulmonary valve replacement for tetralogy of Fallot. Criteria for exclusion included systematic reviews, meta-analyses, case reports/series, unavailable in full text, not available in English, and data collected before 2002.

Results

**Figure 1:**
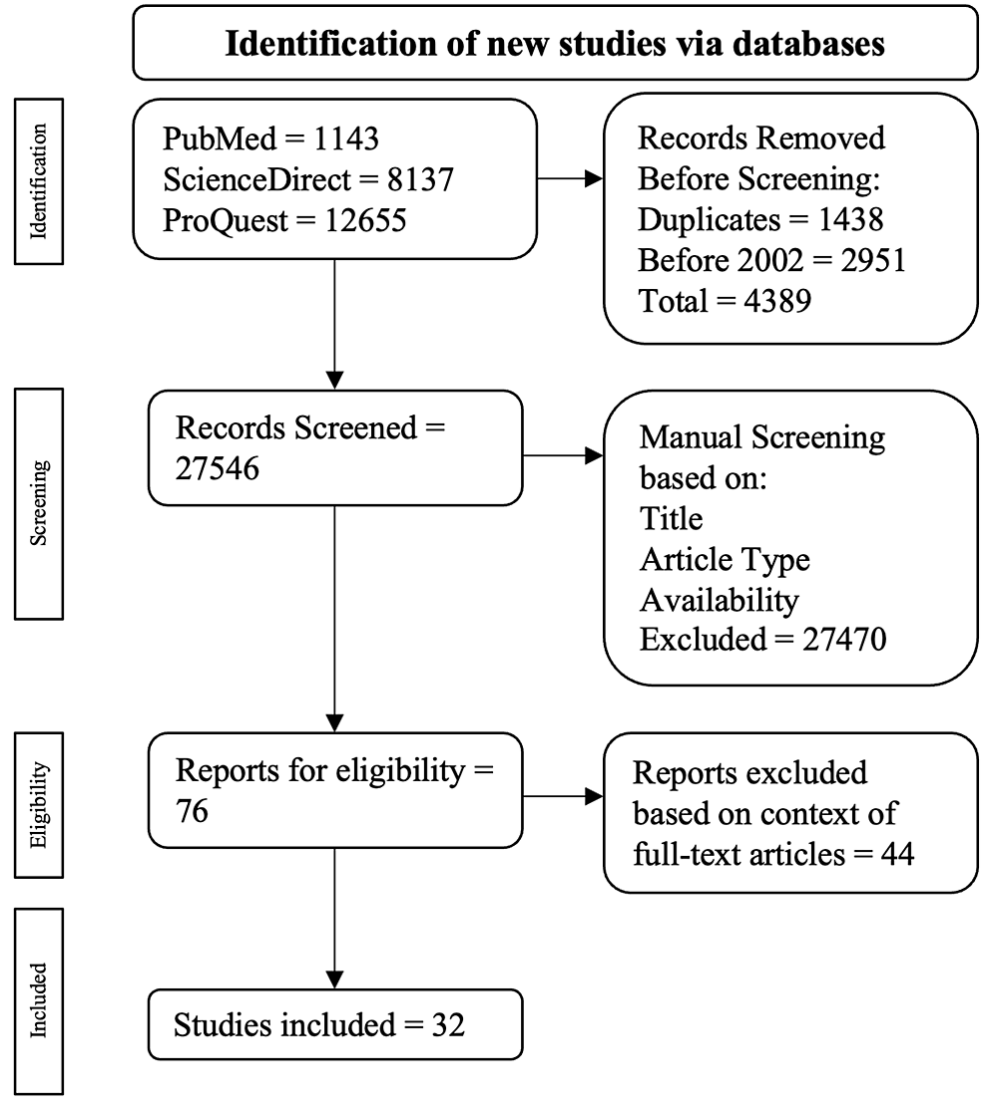
Screening of publications following PRISMA guidelines Ref no- [7]

A total of 21,935 articles were found, with 1,143 from PubMed, 8,137 from ScienceDirect, and 12,655 from ProQuest. Among the exclusions, 1,438 were duplicates, and 2,951 were published before 2002. This resulted in 4,389 being excluded during the automatic screening algorithm, leaving 27,546 articles for manual screening. Articles were manually screened based on title, study type, abstract, and full-text availability. Seventy-six (76) articles were eligible for screening, with 32 publications ultimately included (Figure 1).

Discussion

Indications for Pulmonary Valve Replacement

The reasons for PVR following rTOF are severe PR and severe PR with pulmonary stenosis [8]. There are many indications for why surgery must occur, outlined in Table 1. The PR fraction is significantly associated with needing PVR, especially in asymptomatic patients [9]. Arrhythmias in patients suffering from PR presented as a complete right bundle branch block, followed by a first-degree atrioventricular heart block [10,11]. Patients typically have a QRS duration > 180 ms, Lown 4a/4b class arrhythmia, RV distal PA peak systolic gradient > 30 mmHg, and tricuspid regurgitation grade 1 to 3 [12]. Common symptoms indicating the need for PVR are fatigue and diminished exercise tolerance [11,13]. Oosterhof et al. observed that only higher right ventricular end-diastolic volume (RVEDV) and male sex were found to be significant predictors for a long preoperative QRS duration [14].

**Table 1:** Articles focusing on the indications for PVR after the initial rTOF regarding PVR timing PVR - Pulmonary Valve Replacement; RVESVI - Right Ventricular End-Systolic Volume Index; RV - Right Ventricule; rTOF -Repaired Tetralogy of Fallot; RVEDVI - Right Ventricular End-Diastolic Volume Index; LV - Left Ventricle; PR - Pulmonary Regurgitation; PRF - Pulmonary Regurgitation Fraction.

	Author	Country	Design & Study Population	Assessment Measures	Findings	Conclusion
1	He et al., 2019 [18]	China	Case-Control Study (n = 81)	Imaging studies 6 months after PVR or recruitment.	The PVR group had a lower adverse effect rate, with RVESVI being an independent predictor of RV size normalization.	PVR can reduce RV size and, thus, preserve function. RVESVI thresholds should be updated to >120 mL/m^2^.
2	Borowski et al., 2004 [12]	Germany	Retrospective Cohort Study (n = 18)	Pre- and post-PVR EKG 24-hour Holter monitor. Imaging studies pre- and post-PVR. Cardiac catheterization pre- and post-PVR	The average time from rTOF to PVR was 18.5 years. Patients who were older than 5 when they underwent rTOF had long redo-free intervals. The mean RVEDVI decreased by 30.3%.	PVR proves to be effective in reducing diastolic dimensions of dilated RV.
3	De Ruijter et al., 2002 [11]	USA	Retrospective Cohort Study (n = 171)	Imaging, cardiac catheterizations, and EKG 24-hour Holter recordings were used pre- and post-PVR.	The average age of correction was 9.2 years. Patients who received a transannular patch had a higher incidence of right ventricular dilation compared to those with a transatrial patch, as well as a longer QRS duration.	There are no clear indications for pulmonary valve replacement, as exploratory tests don’t always correlate with clinical conditions.
4	Spiewak et al., 2020 [9]	Poland	Retrospective Cohort Study (n=61)	Cardiac magnetic resonance pre- and post-PVR	The ratio of RV to LV volume and PR fraction showed an acceptable capacity to distinguish between patients needing PVR and those being treated conservatively.	The RV/LV ratio and PRF were substantially associated with the requirement for PVR after rTOF.

PR severity was not correlated with indexed right ventricular end-systolic volume (RVESVi), RVEDV, baseline right ventricular ejection fraction (RVEF), or postoperative decreases in right ventricle end-systolic volume (RVESV) or RVEDV [15]. Pre-operatively, patients had severe right ventricle (RV) dilatation and moderate to severe PR [16]. 69% of physicians relied on RVEDV to determine whether a referral was needed for a PVR. The left ventricular end-systolic volume index (LVESVi) was elevated, but the left ventricular end-diastolic volume index (LVEDVi) was within the normal range [16]. In asymptomatic patients, a combination of RVESV up to 90 ml/m2 with an RVEDV up to 170 ml/m2 was associated with a favorable remodeling [17]. Correlation between PVR and preoperative right ventricular end-systolic volume index (RVESVi) has shown benefits in asymptomatic repaired ToF (rToF) patients, including RV size and function and normalization of size in those with moderate or significant pulmonary regurgitation [18]. It's also crucial to note that RV/LV ratio is significantly associated with the need for PVR, especially in asymptomatic patients [9].

New York Heart Association (NYHA) Class Changes Following Pulmonary Valve Replacement

Patients had either moderate or severe PR before surgery, and all had immediate improvement following PVR [19]. Egbe et al. observed that in their cohort, 82.6% were dismissed home, 1% to other hospitals, 2.8% to nursing facilities, and 13.6% to home health care [20]. NYHA class before surgery was an independent risk factor for death following PVR [21]. Rotes et al. found that the mean NYHA class decreased from 2.2 to 1.2 following a median follow-up of 8 months. Early postoperative NYHA class II was linked with RV peak systolic strain [22]. The NYHA class at follow-up was unrelated to any other factors, such as prior palliation operation, QRS length, age at the time of PVR, including LV peak systolic strain, age at initial repair, 3+ previous cardiac surgical procedures, or RV size [22].

Changes in Arrhythmias and QRS Following Pulmonary Valve Replacement

Arrhythmias observed before surgery tended to persist following the operation, and publications found can be listed in Table 2 [11]. Patients with preoperative atrial or ventricular arrhythmias that required treatment did not significantly improve [23]. QRS intervals did vary in how they changed following surgery. QRS duration, mean pulmonary gradient, and the mean RV did not differ much between pre- and post-PVR procedures [17,24-26]. Other studies have found that the QRS decreased significantly following PVR [8,14,27]. Aleligne et al. observed that irrelevant of the type of procedure, there was a 6 ms decrease in the QRS duration in patients whose QRS was over 140 ms three years following surgery [27]. After surgery, the mean QRS duration decreased by 7.4 ms on average, and patients with a preoperative QRS of > 150 ms experienced a significant reduction in QRS [14]. Romeo et al. also found that older age at the correction and a more extended period between TOF correction and PVR were independently and significantly associated with the progression of QRS duration, such as bundle branch blocks [28]. Scherptong et al. observed that severe QRS prolongation is a significant risk factor for adverse outcomes in long-term follow-up patients following ToF repair [29].

**Table 2:** Articles focusing on arrhythmia changes following PVR after the initial rTOF regarding PVR timing RV: Right Ventricle; PVR: Pulmonary Valve Replacement; TOF: Tetralogy of Fallot

	Author	Country	Design & Study Population	Findings	Conclusion
1	Aleligne et al., 2019 [27]	USA	Retrospective Cohort Study (n = 85)	The QRS at the 3-year follow-up was decreased by 6 ms, and median RV size also decreased significantly at the 3-year follow-up 7.3%	PVR in patients with high-risk TOF leads to a reduction in the QRS and median RV size 3 years following surgery.
2	Oosterhof et al., 2007 [14]	Netherlands	Retrospective Cohort Study (n = 99)	Patients with a preoperative QRS duration of less than 120ms had no significant change in duration after surgery. Patients with a preoperative QRS duration of 150-180 or greater had a slight shortening of duration after surgery.	Initially, surgery resulted in a decrease in QRS duration in patients with preoperative QRS > 150ms, but over time, there was a steady increase.
3	Yun et al., 2018 [8]	Korea	Cohort Study (n = 67)	Following PVR, the cardio-thoracic ratio decreased by 3.4%, and QRS duration significantly decreased by 6.3 ms.	Following a PVR, cardio-thoracic ratio and QRS durations were reduced. Patients with right ventricular outflow tract hypokinesis may not have their QRS duration decreased below 180 ms after PVR.
4	Romeo et al., 2020 [28]	Netherlands	Prospective Cohort Study (n = 158)	PVR was performed at a mean age of 28 and 23.4 years after correction. Survival was 98.1% post-PVR. Women, on average, had shorter QRS duration after PVR. The longer time period between correction and PVR was associated with an increased risk of cardiac death.	Prolongation of QRS duration after PVR was associated with a longer time between correction and PVR. Prevention of progressive QRS prolongation by earlier PVR can potentially reduce the hazard of adverse events after PVR.
5	Scherptong et al., 2010 [29]	Netherlands	Prospective Cohort Study (n = 90)	Patients with a pre-operative QRS duration >180 ms had a 76% event-free survival rate at 5 years, while patients with a QRS duration <180 ms had a 90% survival rate.	Major predictors of unfavorable outcomes during long-term follow-up of patients with TOF include severe QRS prolongation, either before or after PVR, and the absence of a reduction in QRS duration after PVR.

Ventricle Volume Changes Following Pulmonary Valve Replacement

Patients with enlarged right atrium (RA) before PVR had reduced functional capacity and experienced more composite adverse events in the follow-up [24]. Indexed RA area decreased after PVR with a corresponding decrease in the prevalence of RA dilation [25]. An average reduction of 25% in RA volume and a 6% reduction in RA diameter [24,30]. Before and after PVR, there was no relationship between chamber size and contralateral ventricular function. Although there was no correlation between pulmonary regurgitation severity and LV size or function, patients with higher levels of pulmonary regurgitation had larger right ventricles both before and after surgery [16,22]. Overall, the right ventricle (RV) size improved significantly following PVR, with a significant decrease in size [23,30,31]. Heng et al. observed that 70% of patients achieved normalization of RV volumes [25].

Following PVR, there was a significant improvement in RVEDV/ESV and LVEDV/ESV, with a significant reduction in RVEDV shown in Table 3 [10,19,32]. Quail et al. observed that the RV of 64.7% of patients completely normalized, but in most patients, RVSV and RVCO remained unchanged [32]. The mean RVEDVi and RVESVi volume difference significantly reduced following PVR [13,16,24,25,31]. Quail et al. observed that the tendency for total normalization following PVR declined as the RVEDVi size increased [32]. Yim et al. observed an increase in LVEDVi following PVR [26]. Contrary to the results described, Heng et al. observed that both LVEDVi and LVESVi reduced immediately following surgery, while Tobler et al. observed that LVEDVi and LVESVi did not change [25,33].

**Table 3:** Articles focusing on ventricle changes such as dilation and end/systolic volume changes following PVR after the initial rTOF regarding PVR timing RVEDVI - Right Ventricular End-Diastolic Volume Index; RVESVI - Right Ventricular End-Systolic Volume Index; PVR - Pulmonary Valve Replacement; RA - Right Atrium; RV - Right Ventricle; EDFF - End-Diastolic Forward Flow; RVEDV - Right Ventricular End-Diastolic Volume; RVESV - Right Ventricular End-Systolic Volume; RVOT - Right Ventricular Outflow Tract; RVEF - Right Ventricular Ejection Fraction; EF - Ejection Fraction; LV - Left Ventricle; TOF - Tetralogy of Fallot; LVESVI - Left Ventricular End-Systolic Volume Index.

	Author	Country	Design & Study Population	Findings	Conclusion
1	Ait-Ali et al., 2020 [24]	Italy	Retrospective Cohort Study (n = 41)	RVEDVI and RVESVI decreased significantly after PVR by 38% and 38.8%. Patients who had adverse outcomes tended to be older, worse VO2/kg/min, higher Nt-ProBNP, more dilated RA, and more significant tricuspid regurgitation.	RV volume and function may not be the only role in deciding whether to undergo PVR, as RA dimension variations have led to adverse outcomes.
3	Pijuan-Domenech et al., 2014 [30]	Spain	Prospective Cohort Study (n = 20)	Right ventricular diastolic and systolic volume decreases were found in all cases, with a mean of 35%. Right atrial four-chamber echocardiographic measures and volumes, as well as pulmonary EDFF, significantly decreased.	Following PVR, right ventricular diastolic metrics and right atrial volumes improved in line with the known decrease in RV volumes.
4	Heng et al., 2017 [25]	UK	Prospective Cohort Study (n = 57)	Significant reductions were in the RVEDVi and RVESVi at 33.2% and 32.5%, respectively. Normal RVEDV and RVESV were achieved in most patients < 158 ml/m2 and <82 ml/m2, respectively.	Significant right heart structural reverse remodeling takes place immediately after PVR. Undergoing a PVR before RVEDVi reaches 82mL/m2 gives an optimal chance of restoring RV function.
5	O’Meagher et al., 2014 [16]	Australia	Prospective Cohort Study (n = 18)	A significant reduction in RVEDVi by 38.7% was observed following PVR. There was a negative correlation between large pre-PVR RVOT volumes and post-surgical RVEF.	Normalization of RV volume is unlikely to be achieved above a pre-PVR right ventricular end-diastolic volume index of 165 mL/m2 or more.
7	Burkhardt et al., 2017 [34]	USA	Retrospective Cohort Study (n = 10)	RV and LV EF remained constant. There was no change in LV circumferential strain before and after PVR, as well as LV torsion. There was also no difference in time to measure peak LV circumferential strain.	PVR allows for the remodeling of ventricle volumes for TOF patients who experience significant pulmonary regurgitation.
8	Henkens et al., 2007 [15]	Netherlands	Prospective Cohort Study (n = 27)	RVESVi pre-PVR was the best predictor of RVEDVi and RVSESVi following surgery. The severity of pulmonary regurgitation was not related to RV dimensions or function before or after PVR in patients who underwent CMR.	Indexed RVESV and corrected RV EF were found to better predict indexed RVESV and RVEDV after PVR and RVEF after PVR, respectively.
9	Quail et al., 2012 [32]	UK	Cohort Study (n = 87)	Most patients experienced "normalization" of RVEDV and RVESV after PVR. There was a considerable improvement in resting cardiac output.	Individuals with moderate RV dilatation and severe PR have a low probability of experiencing significant short-term progression. This can help determine the interval between CMR exams and the best time to do a future PVR.
10	Sabate Rotes et al., 2014 [21]	Spain	Retrospective Cohort Study (n=133)	For both the left and right ventricles, the longitudinal peak systolic strain and the strain rate before PVR were modest and did not change after surgery. Patients with superior preoperative LV and RV peak systolic strain were found to have better postoperative LV and RV peak systolic strain.	Patients with repaired tetralogy of Fallot who underwent PVR had lower LV and RV systolic and diastolic deformational characteristics, and there was no significant change after surgery.
11	Tsang et al., 2010 [13]	China	Retrospective Cohort Study (n = 16)	The mean RVEDVI decreased significantly after pulmonary valve replacement by 40.1%. The right ventricular ejection fraction and maximum oxygen consumption improved after the operation, rising by 5% and 7.4%, respectively.	It is safe to replace a pulmonary valve for severe pulmonary regurgitation after tetralogy of Fallot repair. To determine the time of pulmonary valve replacement more precisely in this patient population, more study is required.
12	Yim et al., 2016 [26]	USA	Retrospective Cross-Sectional Study (n = 50)	LV and RV longitudinal strain was reduced early post-operatively, followed by recovery of biventricular systolic strain. Cardiac magnetic resonance RVEDVi and RV volume corrected with RV strain pre-op.	The effect of PVR on long-term RV adaptation points to implications for determining optimal timing for intervention.
13	Graham et al., 2018 [23]	USA	Retrospective Cohort Study (n = 93)	PVR was associated with low mortality, an 81% decrease in RV size, and an increase of 59% in ability index with a durability of 11 years for the valves.	PVR is associated with low mortality, a decrease in RV size, and an increase in ability index.
14	He et al., 2022 [31]	China	Retrospective Cohort Study (n = 42)	There was a significant reduction in RVEDVI and RVESVi by 38.7% and 43.9% after PVR. Adverse outcomes were more likely to occur in patients with lower LVESVi. Freedom from adverse events at 3 years was 88.1% and 58.2% at 5 years.	There was a significant reduction in RV volume early after PVR, followed by further improvement.

There is also a marginally decreased RV diastolic strain rate, but RV peak systolic strain before surgery was independently linked with better RV peak systolic strain after surgery [22]. However, Burkhardt et al. observed that LV circumferential strain, torsion, and time to peak circumferential strain did not differ before and after PVR [34]. Better LV peak systolic strain before surgery was linked to better LV peak systolic strain after surgery, with strain falling early in the post-PVR period [22,26].

Ventricle Function and Ejection Fraction Changes Following Pulmonary Valve Replacement

As anticipated, a significant reduction in pulmonary regurgitation caused the right-sided chambers to change and the RV's systolic function to improve [22,24]. Articles mostly observing a change in EF can be found in Table 4. Effects of PVR on RV ejection fraction (RVEF) varied between research. Ait-Ali et al. observed that RVEF remained unchanged following PVR [24]. Heng et al. found that the RVEF decreased by 12% post-PVR but normalized to baseline preoperatively by midterm follow-up [25]. Two studies found a significant increase in REVF following PVR [16,18]. This may be due to the right ventricular outflow tract akinetic length on cardiac magnetic resonance decreasing immediately after PVR, which was sustained at midterm follow-up [25]. There is an inverse relationship between pre-PVR linear right ventricular outflow tract scar length and change in biventricular ejection fractions [25].

**Table 4:** Articles focusing on the change of function and EF of ventricles following PVR after the initial rTOF regarding PVR timing RVEDV - Right Ventricular End-Diastolic Volume; RVESV - Right Ventricular End-Systolic Volume; PVR - Pulmonary Valve Replacement; RVEF - Right Ventricular Ejection Fraction; LVEDV - Left Ventricular End-Diastolic Volume; LVESV - Left Ventricular End-Systolic Volume; LVEF - Left Ventricular Ejection Fraction.

	Author	Country	Design & Study Population	Findings	Conclusion
1	Chalard et al., 2012 [10]	France	Retrospective Cohort Study (n = 21)	RVEDV and RVESV significantly decreased by 42.7% and 40.8% following PVR without improvement of RVEF LVEDV and LVESV after PVR decreased significantly by 50% and 57.1%, respectively, leading to an increase in LVEF.	There is significant improvement in LVEF following PVR that is associated with pre-PVR RVEDV
2	Tobler et al., 2012 [33]	Canada	Retrospective Cohort Study (n = 39)	LVEF significantly improved by 4% following PVR. In patients with poor LVEF prior to surgery, LVESV decreased by 16.9% after PVR, but LV end-diastolic volumes were not significantly changed.	PVR appears to have a beneficial influence on postoperative LVEF and LVESV.

Different studies mainly agreed upon the change in the LV function and LVEF. The mean LVSVs and CO at rest were comparable, and there were no discernible variations in LV volume between the operated and unoperated groups [32]. Quail et al. found that after surgery, there were significant improvements in LV function, with increases in LVSV and LVCO of 19% and 16%, respectively [32]. Chalard et al. observed an increase in EF, which was thought to be due to a decrease in both LVEDV and LVESV [10]. Tobler et al., Rotes et al., and Heng et al. all found an increase in LVEF, while Tobler et al. specifically observed that PVR improved LVEF more for patients with lower preoperative LVEF and higher preoperative LVESVi [22,25,33]. However, Yim et al. observed that even with an increase in LVEDVi following PVR, there is no significant difference in LVEF or RVEF [26].

Other Important Outcomes Following Pulmonary Valve Replacement

Warner et al. observed that 16.7% of patients who underwent pre- and post-op exercise testing significantly increased peak workload 36.7 months after surgery [19]. 97.2% of patients reported symptomatic improvement 80.6 months following surgery [19]. There was also a significant decrease in cardiomegaly (cardiothoracic ratio) following PVR [25,35]. This is consistent with a significant reduction in LnNt-ProBNP and a disappearance of common symptoms [11,24].

The in-hospital mortality rate was 1.5%, with a free from PVR reintervention rate of 75% at 15 years [20]. Independent risk factors for mortality were older age at repair, > 2 previous cardiac surgeries, worse VO2/kg/min, more dilated RA, NYHA class III/IV before PV, and a large body surface area [21,24]. The articles can be found in Table 5.

**Table 5:** Articles focusing on other outcomes not mentioned following PVR after the initial rTOF regarding PVR timing PR - Pulmonary Regurgitation; TOF - Tetralogy of Fallot; CT - Cardiothoracic Ratio; RVEDDI - Right Ventricular End-Diastolic Diameter Index; LV - Left Ventricle; PVR - Pulmonary Valve Replacement; PV - Pulmonary Valve; NYHA - New York Heart Association; SCD - Sudden Cardiac Death; VT - Ventricular Tachycardia; RVEDVI - Right Ventricular End-Diastolic Volume Index; RV - Right Ventricle; CMR - Cardiac Magnetic Resonance.

	Author	Country	Design & Study Population	Findings	Conclusion
1	Warner et al., 2003 [19]	USA	Retrospective Cohort Study (n = 36)	Patients had clinical improvements in exercise capacity. Peak improvement workload. There was a 30% reduction in the RVEDDI.	Timely insertion of a competent pulmonary valve in children, adolescents, and young adults with significant PR after TOF repair results in subjective and objective improvement in exercise capacity and is associated with a reduction in right ventricle size.
3	Shiokawa et al., 2012 [35]	Japan	Retrospective Cohort Study (n = 19)	The CTR significantly improved by a reduction of 4.8%. The percentage of the LV that is ejected significantly increased by 10.4%. The QRS duration significantly decreased by 19.6 ms.	Replacement of the pulmonary valve decades after the prior TOF was repaired produced therapeutic advantages and low mortality.
4	Egbe et al., 2019 [20]	USA	Retrospective Cohort Study (n = 18,353)	The median age was 34 years, with a 1.5% in-hospital mortality following PVR. However, there has been an increase in PVR, with a decrease in age at surgery and in-hospital mortality.	Despite the increase in PVR admissions, there was a decrease in in-hospital mortality and age at the time of PVR.
5	Sabate Rotes et al., 2014 [22]	USA	Retrospective Cohort Study (n = 278)	Independent risk factors for mortality were older age at repair. 15 years free from PV reintervention was 75%.	Important prognostic markers include the overall number of cardiac surgeries, the date of the surgery, and the NYHA classification.
6	Pastor et al., 2020 [37]	USA	Cross-Sectional Study (n = 189)	Primary outcome of death, SCD, sustained VT, or NYHA class > III. The rates of five and ten years without an occurrence were 97% and 91%, respectively. The composite result and post-PVR RVEDVi were not related.	PVR-induced mild or moderate RV dilation, as assessed by CMR-derived RVEDVi, was not related to worse outcomes.

Timing of Pulmonary Valve Replacement

The average age of patients undergoing PVR varied significantly between studies; those acquired can be found in Table 6. Certain studies focused on the time of PVR following ToF repair compared to the patient's actual age at the time of PVR. Lim et al. found that PVR was performed on average 8.3 years after ToF repair [36]. Borowski et al. observed that the average time to PVR following ToF repair was 18.5 years, possibly due to the onset of severe PR grade 3 being 11.8 years [12]. Two studies observed that PVR surgery was performed an average of 23-26 years following the initial ToF repair [16,23,28]. The actual mean age of surgery had variations but was not as drastic as the time of PVR following ToF repair. Several studies found that the average age of PVR surgery was 12-16 years [8,26,36]. However, a most recent study by Romeo et al. observed that the mean age of PVR was 28 years [28]. It is important to remember that these age differences can vary due to regional and current testing standard variations, so there may be a need to narrow the time window to perform surgery.

**Table 6:** Articles focusing solely on the possible timing of PVR following the initial rTOF PVR - Pulmonary Valve Replacement; RVESV - Right Ventricular End-Systolic Volume; RVEDV - Right Ventricular End-Diastolic Volume; TOF - Tetralogy of Fallot; RV - Right Ventricle; CTR - Cardiothoracic Ratio.

	Author	Country	Design & Study Population	Findings	Conclusion
1	Dobbels et al., 2017 [38]	Belgium	Retrospective Cohort Study (n = 273)	There is no significant difference in survival between early- and late PVR. Worse event-free survival was observed following early- PVR. However, right ventricular function and morphology were preserved better in patients who had early PVR.	Early PVR led to higher PVR-related morbidity but preserved the right ventricular morphology and function, which may be beneficial in longer-term outcomes.
2	Alvarez-Fuente et al., 2015 [17]	Spain	Retrospective Cohort Study (n = 35)	Patients with an RVESV under 90 ml/m2 combined with RVEDV 170 ml/m2 before surgery had a greater chance of reduction following surgery.	When deciding the optimal timing of PVR in TOF, RVEDV, and RVESV should be considered.
3	Lim et al., 2004 [36]	South Korea	Retrospective Cohort Study (n = 58)	Early PVR led to significant symptomatic improvement and decreased CTR by 6%. The symptomatic group of early PVR was older and, compared to the study mean of 8.3, had a longer interval between repair and TOF.	Earlier PVR in patients with chronic PR should be considered to improve outcomes before worsening RV function and deteriorating functional class in both asymptomatic and symptomatic patients.

There was an association between a composite outcome (worsening NYHA class, death, and further complications) and older age at PVR [37]. Ait-Ali found that by the time PVR was scheduled, 14 patients experienced adverse cardiac events such as atrial and ventricular arrhythmias, and one patient had a worsening NYHA class [24]. Compared to those who survived PVR, very few patients who died at follow-up had worse LV and RV function before surgery. These findings point to the potential use of the new quantitative deformational characteristics to choose the best PVR timing [22].

Patients who underwent early PVR presented with significantly short QRS duration before and after PVR compared to patients who underwent late PVR [38]. The right ventricular size was significantly smaller in patients who underwent early PVR than those who underwent late PVR due to hypertrophy [17,38]. RVEDV, RVESV, RVEDVi, and RVESVi were significantly increased before performing late-PVR, which led to a reduced decrease in those parameters following surgery compared to earlier-PVR [17]. The function of the right ventricle was significantly worse in patients who underwent late PVR compared to early PVR [38]. Earlier PVR in patients with chronic PR should be considered to improve outcomes before worsening RV function and deteriorating functional class in asymptomatic and symptomatic patients [36]. Left ventricular function did not differ between early and late PVR patients [38].

All-cause mortality was not significantly different for patients who underwent early or late PVR [38]. The combined efficacy end-point was not significantly different in patients who underwent early or late PVR [38]. There were no significant differences in survival in patients who had early, late, or no PVR [38]. Patients with early PVR (before age 16) had more reinterventions of the new pulmonary valve than those with late PVR [38].

Bias

All studies were assessed for bias, and the results did vary. A few articles did not disclose their full protocol and weighed on the overall bias. A medium-risk bias combined was determined. The grading of recommendation, assessments, development, and evaluations (GRADE) tool was used to assess the individual risk of bias, which evaluates flaws like indirectness, publications, and imprecision.

## Conclusions

While several factors may affect the outcomes and indications for PVR in patients with ToF, benefits have been found throughout reviewed studies. The main indications for PVR include severe PR and severe PR with pulmonary stenosis, which can cause fatigue and diminished exercise tolerance in patients. Due to the pre-operative findings in patients with severe right ventricular dilatation and moderate to severe PR, the majority of physicians found that relying on RVEDV was necessary to consider in decision-making regarding PVR referrals. In asymptomatic patients, the correlation between PVR and preoperative RVESVi was found to show benefits, including RV size, function, and normalization of size in those with moderate or significant PR. These patients were found to have immediate improvement post-PVR.

After PVR, the majority of patients' RV size was completely normalized. However, patients with preoperative atrial or ventricular arrhythmias that required treatment did not significantly improve. Correction at an older age and a longer period between ToF correction and PVR were independently and significantly associated with the progression of QRS duration. Symptomatically, almost all patients reported improvement immediately and months after surgery. It was observed that a few of the patients who underwent pre- and post-operative testing had significantly increased peak workload after surgery. Regarding LV function, better LV peak systolic strain before surgery was linked to strain falling early in the post-PVR period. The change in LVEF was mainly agreed upon across different studies.

Across many studies, the average ages of patients varied greatly regarding the timing of PVR, which can range due to various factors both regionally and due to testing standards. This may call for the necessity of a narrower time window to perform PVR to improve outcomes for ToF patients. Early PVR patients presented with significantly shorter QRS duration before and after surgery, and it was also found that these patients who underwent early PVR had significantly smaller RV sizes than those who underwent surgery later. RV function in patients who underwent late PVR was found to be significantly worse than those who were operated on earlier. These findings point to the potential of using new quantitative deformational characteristics to choose the best timing for PVR to facilitate better outcomes for ToF patients.
